# Axl Regulation of NK Cell Activity Creates an Immunosuppressive Tumor Immune Microenvironment in Head and Neck Cancer

**DOI:** 10.3390/cancers17060994

**Published:** 2025-03-15

**Authors:** Kourtney L. Kostecki, Regan L. Harmon, Mari Iida, Madelyn A. Harris, Bridget E. Crossman, Justine Yang Bruce, Ravi Salgia, Deric L. Wheeler

**Affiliations:** 1 Department of Human Oncology, School of Medicine and Public Health, University of Wisconsin, Madison, WI 53705, USA; klkostecki@gmail.com (K.L.K.); rlharmon2@wisc.edu (R.L.H.); iida@humonc.wisc.edu (M.I.); maharris7@wisc.edu (M.A.H.); bridget.crossman@wisc.edu (B.E.C.); 2 Department of Medicine, School of Medicine and Public Health, University of Wisconsin, Madison, WI 53705, USA; jybruce@medicine.wisc.edu; 3 Carbone Cancer Center, University of Wisconsin, Madison, WI 43792, USA; 4 Department of Medical Oncology and Therapeutics Research, City of Hope Comprehensive Cancer Center, Duarte, CA 91010, USA; rsalgia@coh.org

**Keywords:** head and neck cancer, Axl, NK cells, immunosuppression, tumor microenvironment

## Abstract

*Background*: Head and neck cancer (HNC) evades immune responses by manipulating the tumor immune microenvironment (TIME). Tumor-bound Axl has been implicated in promoting an immunosuppressive TIME in HNC, though its precise role remains unclear. Understanding Axl’s contribution to immune evasion in HNC could lead to the identification of new therapeutic targets; therapies directed at these targets could be combined with and thereby enhance immunotherapies. *Results:* Using Axl knockout (Axl KO) cell lines derived from the immunologically “cold” MOC2 mouse model, we found that Axl loss delayed tumor growth in immunocompetent mice. This was accompanied by reduced immunosuppressive cells, including MDSCs, T_reg_s, B cells, and neutrophils, and increased infiltration of cytotoxic CD8 T cells and NK cells. To identify the immune population(s) responsible for these changes, Axl KO tumors were implanted in immune-deficient mice. Axl KO tumor growth in athymic nude mice (which lack T cells) was unchanged, whereas tumor growth in NCG mice (which lack NK cells) was rescued, suggesting that NK cells mediate the Axl KO tumor growth delay. Further, Axl loss enhanced NK cell cytotoxicity in vitro and in vivo, and NK cell depletion reversed delayed Axl KO tumor growth. Mechanistically, Axl KO tumors showed decreased expression of CD73 and CCL2, which inhibit NK cells, and increased expression of CCL5 and CXCL10, which promote NK cell recruitment and activation. *Conclusions:* These novel findings suggest that tumor-bound Axl fosters an immunosuppressive TIME by inhibiting NK cell recruitment and function, thereby promoting tumor growth. Targeting Axl may enhance NK cell-mediated tumor killing and improve immunotherapy efficacy in HNC.

## Simple Summary

Head and neck cancer (HNC) is an aggressive disease that often evades the immune system, making it difficult to treat. This study investigates how Axl, a protein overexpressed on tumor cells, contributes to the immune escape strategies of HNC by decreasing the number of immune cells within the tumor. Using mouse models, we show that removing Axl slows tumor growth by reducing the number of immune-suppressing cells and increasing immune cells that can kill tumors, such as natural killer (NK) cells. We found that Axl prevents NK cells from working properly by modulating signals that attract, activate, or inhibit them. By blocking Axl, tumors are more vulnerable to immune-mediated killing. These findings suggest that targeting Axl could improve treatments for HNC by increasing immune cell invasion within the tumor, providing a new way to enhance the effectiveness of immunotherapy.

## 1. Introduction

Head and neck cancer (HNC) impacts hundreds of thousands of people worldwide, with approximately 66,500 new cases and 16,000 associated deaths reported annually in the United States alone [1]. This highly aggressive and heterogeneous disease originates from various sites within the aerodigestive tract, including the oral cavity, larynx, pharynx, and salivary glands. HNC is linked to risk factors such as alcohol and tobacco use or human papillomavirus (HPV) infection [2]. While the current standard-of-care treatments—surgery, radiation, chemotherapy, and immunotherapies [3,4]—are effective for early-stage HNC, outcomes for advanced-stage disease remain poor. The five-year survival rate for late-stage HNC is less than 50%, underscoring the urgent need for improved therapeutic strategies [1].

The progression of solid-tumor cancers, including HNC, is influenced by multiple factors, with one of the most critical being the tumor immune microenvironment (TIME) [5,6]. The TIME consists of diverse cell types, including immune cells, fibroblasts, endothelial cells, and other stromal cell types, all of which can either support or suppress tumor growth [6]. The TIME is often characterized by its immunological “temperature”: tumors described as “hot” have higher levels of pro-inflammatory immune infiltrate, while “cold” tumors have lower levels of pro-inflammatory immune infiltrate. This distinction is particularly significant in predicting responses to immunotherapy. Immunologically hot tumors, such as non-small cell lung cancer and melanoma, tend to have higher response rates to immunotherapy treatments [6,7,8,9,10,11]. In contrast, relatively cold tumors, including HNC, have lower response rates to these therapies [12]. Therefore, elucidating the mechanisms by which cancer cells establish an immunologically “cold” microenvironment, and, more importantly, identifying strategies to reprogram this phenotype into an immune-activated state, are essential for the development of effective immunotherapeutic approaches.

HNC cells employ various strategies to manipulate the TIME to their advantage [13]. In this study, we investigate the role of the receptor tyrosine kinase (RTK) Axl, also known as tyrosine-protein kinase receptor UFO [14], in shaping a cold TIME in HNC. Axl, a member of the TAM family of RTKs (Tyro3, Axl, and MerTK), has been extensively studied for its tumor-intrinsic mechanisms in driving HNC progression [15,16,17]. Axl is often overexpressed in HNC as compared to normal oral tissue; one study found Axl expression in 82% of HNC patient-derived xenografts (PDXs) [15]. Although RTK signaling in general has been found to be enriched in HNC tumors from patients non-responsive to chemotherapy and radiation therapy [18], Axl signaling in particular is implicated in promoting cancer cell proliferation, survival, metastasis, and resistance to chemotherapy [15,16,17,19]. Moreover, recent works have implicated Axl in promoting an immunosuppressive TIME and conferring resistance to immune checkpoint inhibitors (ICIs) in various solid tumors [20,21,22]. Genetic or molecular inhibition of Axl in mouse models of breast [20], ovarian [23], and pancreatic cancers [24] has demonstrated an enhanced anti-tumor immune response and improved efficacy of anti-programmed death 1 (PD1)/programmed death ligand 1 (PD-L1) ICIs. Although Axl has been found to be a viable therapeutic target to inhibit cancer progression and metastasis in HNC [25], there have been no studies evaluating its effects on the TIME and immunosuppressive potential in HNC when inhibited on its own.

Previous work from our laboratory has demonstrated the effect of pharmacological inhibition of TAM family signaling on tumor progression and changes within the TIME in HNC [19,21]. Disruption of Axl and MerTK signaling with the small molecule inhibitor INCB081776 [26] slowed HNC tumor growth, led to the development of a pro-inflammatory TIME, and enhanced tumor response to immunotherapies [21]. This inhibition significantly influenced several immune cell populations within the TIME, including T cells, natural killer (NK) cells, neutrophils, and macrophages [21]. However, this study used a dual Axl and MerTK inhibitor. The focus of this paper is to understand the contributions of tumor-bound Axl alone. By studying Axl on its own, we can better understand how its expression influences the recruitment and activity of specific immune cell populations within the TIME in HNC.

In this novel study, we investigate our hypothesis that Axl contributes to an immunosuppressive TIME in HNC using a series of Axl knockout (KO) cell lines created in a mouse model of HNC. We demonstrate that Axl-mediated exclusion of NK cells from the TIME contributes to the creation of an immunosuppressive environment and enhanced tumor growth.

## 2. Materials and Methods

### 2.1. Cell Lines

The mouse oral cancer 2 (MOC2) cell line was obtained from Dr. Ravindra Uppaluri (Dana-Farber Cancer Institute, Boston, MA, USA) and maintained as previously described [21].

Knockout (KO) cell lines were developed using CRISPR-Cas9 ribonucleoprotein (RNP) technology. Briefly, MOC2 cells were transfected with Cas9-guide RNA (gRNA) complexes using the Lipofectamine CRISPRMAX Transfection Reagent (Invitrogen, Waltham, MA, USA), TrueCut Cas9 Protein v2 (Invitrogen, Waltham, MA, USA), and two TrueGuide Synthetic gRNAs (Invitrogen, Waltham, MA, USA) targeting mouse Axl (#CRISPR468314_SGM, “gRNA-314” and #CRISPR468318_SGM, “gRNA-318”) according to manufacturer instructions. After incubating for 48 h at 37 °C, 5% CO_2_, cells were collected, and single cells were sorted via FACS into individual wells of a 96-well tissue culture plate. Clones were expanded and screened for Axl expression via immunoblot, genotyping, and sequencing analyses.

### 2.2. Genotype Analysis

Cell lines were plated in a 24-well plate, allowed to grow until ~70% confluent, and harvested. The cell pellet was incubated in 50 mM NaOH for 10 min at 95 °C, followed by the addition of 1 M Tris-HCl. Using Terra PCR Direct reagents (Takara Bio, San Jose, CA, USA), polymerase chain reaction (PCR) was performed on this lysate to amplify a region (~750 base pairs) surrounding the gRNA-314 target site. PCR cleanup was performed (IBI Scientific, Dubuque, IA, USA), and the DNA was then quantified using the SpectraMax Quant AccuBlue HiRange dsDNA Assay Kit (Molecular Devices, San Jose, CA, USA), SpectraMax i3x (Molecular Devices, San Jose, CA, USA), and a NanoDrop spectrophotometer (Thermo Fisher Scientific, Waltham, MA, USA). Samples were sequenced by Plasmidsaurus (Louisville, KY, USA), and the sequences were aligned using Benchling (San Francisco, CA, USA).

### 2.3. Immunoblot Analysis

Immunoblot analysis was conducted as previously described [27]. Briefly, whole cell protein lysates were collected, quantified, fractionated by SDS-PAGE, transferred to a PVDF membrane, incubated with the appropriate primary and HRP-conjugated secondary antibodies, and then detected with the Radiance Q chemiluminescent substrate (Azure Biosystems, Dublin, CA, USA). Antibodies (Supplemental Table S1) were used according to the manufacturer’s instructions.

### 2.4. Cell Growth Assay

Cells (5 × 10^4^ cells/well) were plated in a 6-well plate and incubated for 24, 48, or 72 h (*n* = 3 wells per time point). Cells were then trypsinized and counted using the Countess Automated Cell Counter (Invitrogen, Waltham, MA, USA).

### 2.5. Cell Proliferation Assay

Two thousand cells per well were plated and incubated for 72 h in a 96-well plate. Cell proliferation was assessed using Cell Counting Kit-8 (Dojindo Molecular Technologies, Rockville, MD, USA) according to the manufacturer’s instructions. In short, media was removed from the plate and replaced with 100 μL/well of CCK-8 solution diluted 1:10 in media. Cells were incubated for an hour before the plate was read using a Versamax Tunable Microplate Reader (Molecular Devices, San Jose, CA, USA).

### 2.6. Flow Cytometry

Cells were collected and stained for flow cytometry as previously described [21]. Briefly, cells were stained with live/dead dye, incubated with Fc Block (Biolegend, San Diego, CA, USA), stained with fluorescent antibodies, washed, permeabilized overnight using the FoxP3/Transcription Factor Staining Buffer Kit (Tonbo Biosciences, San Diego, CA, USA), and stained with fluorescent intracellular antibodies if needed. UltraComp Beads (Invitrogen, Waltham, MA, USA) or Compensation Beads (Biolegend, San Diego, CA, USA) were stained with fluorescent antibodies. Samples were run on an Attune NxT Flow Cytometer (Invitrogen, Waltham, MA, USA) or an Attune Xenith Flow Cytometer (Invitrogen). Analysis was performed in FlowJo (BD Biosciences, Franklin Lakes, NJ, USA) unless otherwise stated. Supplemental Table S2 details the fluorescent antibodies used, and Supplemental Table S3 provides the gating paths used.

#### 2.6.1. Fluorescence Activated Cell Sorting (FACS)

Forty-eight hours after transfection of the Cas9-gRNA complexes, cells were collected and stained as described above. Live single cells negative for Axl were sorted into individual wells of a 96-well tissue culture plate using a BD FACSAria Cell Sorter (BD Biosciences).

#### 2.6.2. Imaging Flow Cytometry

Cells were collected and stained as described above. Samples were run on a Cytek Amnis ImageStream Mk II Imaging Flow Cytometer (Cytek Biosciences, Fremont, CA, USA). Analysis was performed in IDEAS software version 6.2 (MilliporeSigma, Darmstadt, Germany).

### 2.7. RNA Isolation, cDNA Synthesis, and Quantitative Polymerase Chain Reaction (qPCR)

RNA isolation, cDNA synthesis, and qPCR were performed as previously described [21]. TaqMan Fast Advanced Master Mix and TaqMan probes (Thermo Fisher Scientific, Waltham, MA, USA) were used (Supplemental Table S4) to measure gene expression. The levels of gene expression were analyzed using the ΔΔC_T_ method; GAPDH and β-actin were used for normalization.

### 2.8. In Vivo Mouse Experiments

Animal procedures and maintenance were conducted in accordance with the University of Wisconsin—Madison School of Medicine and Public Health IACUC guidelines. Cell lines were inoculated by subcutaneous injection into the dorsal flank of 7–8-week-old female C57BL/6N (Charles River Laboratories, Wilmington, MA, USA), athymic nude (Envigo, Indianapolis, IN, USA), or NCG (Charles River Laboratories, Wilmington, MA, USA) mice. All cells were resuspended in PBS containing Matrigel (50% *v*/*v*, Corning Inc., Corning, NY, USA) before inoculation.

#### 2.8.1. Tumor Dissociation

Tumors were extracted following euthanasia and dissociated as previously described [21]. The cells were then analyzed using flow cytometry as described above.

#### 2.8.2. NK Cell Depletion

To deplete NK cells, mice were treated with 50 μg/mL anti-NK1.1 antibody (#BE0036, BioXCell, Lebanon, NH, USA) every five days, beginning 5 days prior to tumor inoculation, via intraperitoneal (IP) injection.

### 2.9. Immunohistochemistry (IHC)

IHC was performed as previously described [21]. Tumor tissue samples were collected, fixed in 10% neutral buffered formalin, paraffin embedded, and sectioned. IHC was performed as previously described [21]. Briefly, antigen retrieval was performed by heating the sections in either 10 mM citrate buffer (pH 6.0) or 10 mM Tris—1 mM EDTA buffer (pH 9.0). Samples were incubated with antibodies (Supplemental Table S5), which were detected using either the ImmPRESS HRP Goat Anti-Rat or Goat Anti-Rabbit IgG Polymer Detection Kit (Vector Laboratories, Burlingame, CA, USA) in conjunction with 3,3′-diaminobenzidine substrate (Vector Laboratories, Burlingame, CA, USA). Tissues were counterstained with Mayer’s hematoxylin (Thermo Fisher Scientific, Waltham, MA, USA) and examined using an Olympus BX51 microscope. Quantitation of staining intensity was performed as previously described [28] using FIJI version 1.54.

### 2.10. NK Cytotoxicity Assay

Spleens were harvested from female C57BL/6 mice and crushed through a 70 μm cell strainer using the plunger of a 1 mL syringe. After rinsing the cell strainer with PBS, splenocytes were centrifuged, and NK cells were isolated using the MojoSort Mouse NK Cell Isolation Kit (Biolegend, San Diego, CA) according to manufacturer instructions. Simultaneously, cancer cells at ~70% confluency were harvested. To measure NK cytotoxicity, the Cytotoxicity LDH Assay Kit—WST (Dojindo Molecular Technologies, Rockville, MD, USA) was used according to manufacturer instructions for a non-homogenous ADCC assay. Briefly, 1 × 10^4^ cancer cells and/or 1 × 10^5^ NK cells were added to each well of a 96-well plate. After incubating for 4 h at 37 °C, 5% CO_2_, the plate was spun down, and the supernatant was transferred to a new 96-well plate. The LDH amount in each well was measured, and the percent cytotoxicity was calculated according to manufacturer instructions.

### 2.11. Murine Cytokine Array

Cells were plated and incubated for 24 h at 37 °C, 5% CO_2_, after which the culture media was collected. Cytokine secretion analysis was performed using the Mouse Cytokine Array C3 (RayBiotech, Norcross, GA, USA) kit according to manufacturer instructions. Membranes were imaged using an Azure Biosystems C600 imager (Azure Biosystems, Dublin, CA, USA). Relative cytokine intensities were normalized in comparison to control spots and quantified using the ImageJ package “Protein Array Analyzer” version 1.1.c.

### 2.12. Luminex

Cells were plated and incubated for 24 h, after which the culture media was collected. A Mouse ProcartaPlex Mix & Match Panel (Thermo Fisher Scientific, Waltham, MA, USA) was performed according to manufacturer instructions. Samples were read using the Luminex xMAP INTELLIFLEX System and analyzed using the ProCartaPlex Analysis App (Thermo Fisher Scientific, Waltham, MA, USA).

### 2.13. NanoString

Total RNA from plated cells was isolated and quantified as described above. NanoString analysis was performed by the University of Wisconsin (UW)—Madison Translational Research Initiatives in Pathology (TRIP) lab using the Tumor Signaling 360 panel (NanoString Technologies, Seattle, WA, USA).

Tumors were extracted following euthanasia, and small pieces were flash frozen in isopentane. The CryoPrep System (Covaris, Woburn, MA, USA) was used to pulverize the tumor pieces, and total RNA was extracted using the Direct-zol RNA Miniprep Plus Kit (Zymo Research, Irvine, CA, USA). Quantification was performed using a NanoDrop spectrophotometer (Thermo Fisher Scientific, Waltham, MA, USA). NanoString analysis was performed by the UW-Madison TRIP lab using the PanCancer Immune Profiling panel according to the manufacturer’s instructions (NanoString Technologies, Seattle, WA, USA).

### 2.14. Statistical Analysis

Statistical analyses were performed using GraphPad Prism version 10.3.1 (Dotmatics, Boston, MA, USA). Differences were considered significant when *p* < 0.05.

## 3. Results

### 3.1. Development and In Vivo Growth of Axl KO MOC2 Cell Lines

The mouse oral cancer (MOC) models [29] have been extensively characterized: MOC1 and MOC22 are slow-growing and immunogenic, whereas MOC2 is a rapidly growing and immune-evasive cell line [30]. Previous work from our laboratory confirmed that all three cell lines express Axl, with MOC2 expressing higher levels compared to MOC1 or MOC22 [21]. Additionally, in vivo studies demonstrated that MOC2 tumor growth was more significantly impacted by the Axl/MerTK dual inhibitor INCB081776 [21,26]. However, these studies did not determine the individual contributions of either Axl or MerTK. To determine the role of Axl, we genetically deleted the Axl gene and measured the impact on tumor growth and the TIME.

**Figure 1:**
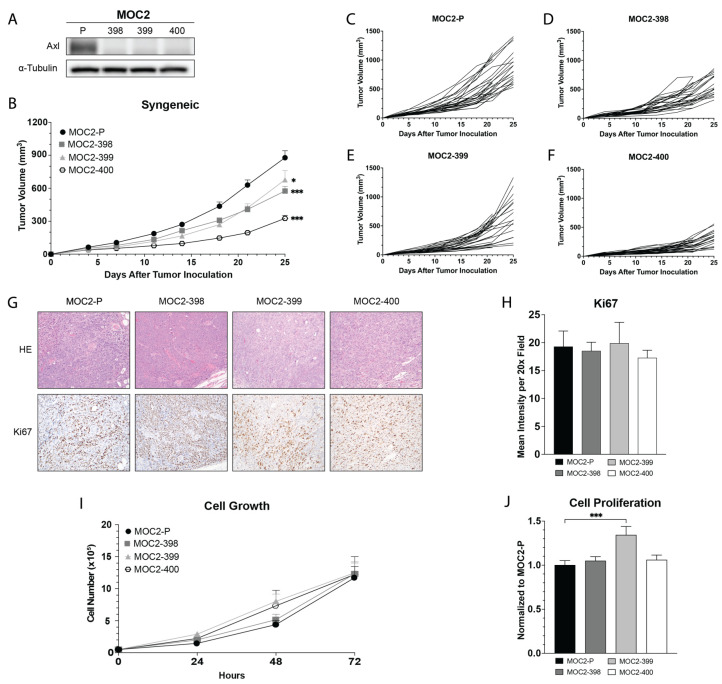
Axl KO results in a tumor growth delay (**A**) Whole cell lysates were harvested and fractionated via SDS-PAGE, followed by immunoblotting for Axl. α-Tubulin was the loading control. The uncropped blots are shown in Supplemental Figure S5. (**B**) Subcutaneous tumors were generated in syngeneic (C57BL/6) mice by inoculating 0.5 × 10^6^ MOC2 parental or Axl KO tumor cells per tumor. Tumor volume was measured twice per week by digital caliper. Mean values and SEMs are shown (*n* = 30 per group). * *p* < 0.05; *** *p* < 0.001. (**C**–**F**) Individual tumor growth rates are shown. (**G**,**H**) Tumors were collected and stained using H&E or IHC. Representative IHC images for each group (20× magnification) are shown. Image quantification was performed. (*n* = 8 tumors per group, *n* = 3 images per tumor). (**I**) Cells (5 × 10^4^) were plated in a 6-well dish and incubated at 37 °C, 5% CO_2_ for the indicated times. Cells were trypsinized, collected, and counted. Mean values and SEMs are shown (*n* = 3 per group). (**J**) Cells were plated into a 96-well plate and incubated at 37 °C, 5% CO_2_ for 72 h. Cell viability was assessed using CCK8. Mean proliferation values relative to MOC2-P and SEMs are shown (*n* = 14 per group). *** *p* < 0.001.

Using the CRISPR/Cas9-ribonucleoprotein (RNP) method with two guide RNAs targeting sequences within the second coding exon of the *Axl* gene, we created three Axl knockout cell lines (Axl KO), derived from single cell clones: MOC2-398, MOC2-399, and MOC2-400 (Figure 1). We first confirmed the loss of Axl protein expression by immunoblot (Figure 1A), flow cytometry (Supplemental Figure S1A,B), and imaging flow cytometry (Supplemental Figure S1C). Genotype analysis confirmed indels near the gRNA-314 target site in all 3 KOs, with average mismatch percentages of 25%, 51%, and 56% in MOC2-398, MOC2-399, and MOC2-400, respectively, compared to the parental MOC2 cell line (MOC2-P) (Supplemental Figure S1D). Immunoprecipitation of Axl followed by an immunoblot further confirmed the deletion of Axl (Supplemental Figure S1E).

To investigate the impact of Axl KO on the TIME in vivo, Axl KO tumor cells were implanted into syngeneic mice. At day 25, tumor growth was delayed in all three Axl KOs compared to MOC2-P, with MOC2-400 exhibiting the most pronounced growth delay (Figure 1B–F). Interestingly, in vivo proliferation rates, as indicated by Ki67 staining of the tumors, did not significantly differ between Axl KO and MOC2-P tumors (Figure 1G,H). Similarly, no substantial differences in cell proliferation or growth were observed in vitro, except for a statistically significant increase in cell proliferation seen in MOC2-399 (Figure 1I,J). Collectively, these findings demonstrate that Cas9/RNP-mediated Axl knockout was successful and led to slower tumor growth in vivo. However, this growth delay does not appear to be driven by changes in tumor cell proliferation rates.

### 3.2. Axl KO Results in a Hotter TIME

**Figure 2:**
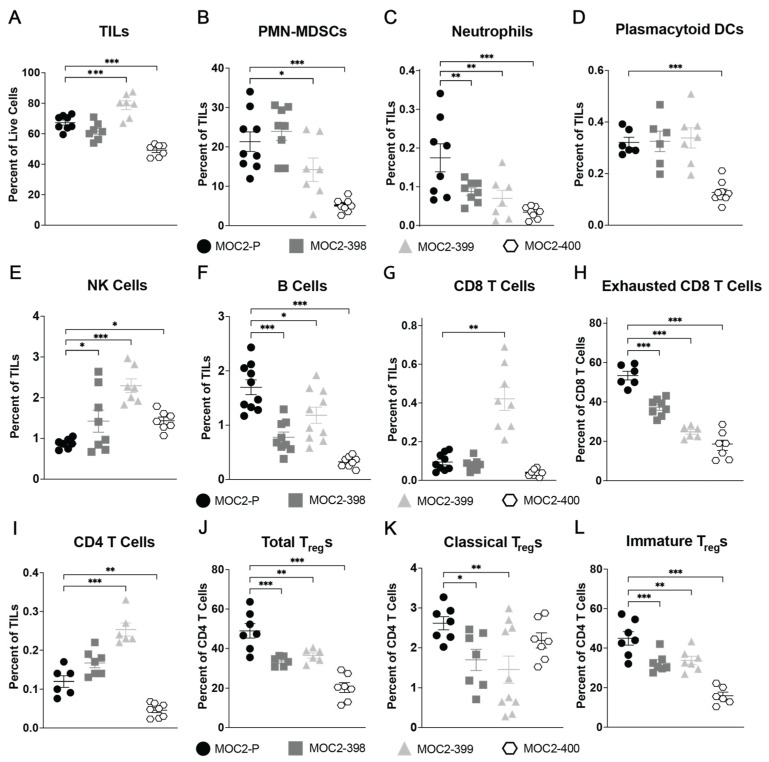
Axl KO results in a hotter TIME. Subcutaneous tumors were generated in syngeneic (C57BL/6) mice by inoculating 0.5 × 10^6^ MOC2 parental and Axl KO cells per tumor. (**A**–**L**) Tumors were collected 21 days after inoculation, and tumor-infiltrating lymphocytes (TILs) were analyzed via flow cytometry. Mean and SEMs are shown (*n* = 6–10 per group). * *p* < 0.05; ** *p* < 0.01; *** *p* < 0.001.

Given that there were no differences in cell proliferation rates between parental MOC2 and the Axl KO tumors, we next investigated whether the observed tumor growth delay in the Axl KO tumors was driven by changes in the TIME. To characterize the immune cell composition, we performed flow cytometric analysis of tumor-infiltrating leukocytes (TILs) in parental and Axl KO MOC2 tumors (Figure 2). Notably, MOC2-400 had significantly lower levels of TILs compared to the other cell lines, while MOC2-399 tumors had increased levels of TILs (Figure 2A). Overall, Axl KO was associated with statistically significant reductions in infiltrating neutrophils (Figure 2C), B cells (Figure 2F), exhausted CD8 T cells (Figure 2H), total regulatory T cells (T_reg_s) (Figure 2J), classical T_reg_s (Figure 2K), and immature T_reg_s (Figure 2L), alongside increases in infiltrating natural killer (NK) cells (Figure 2E). Levels of polymorphonuclear myeloid-derived suppressor cells (PMN-MDSCs) had a significant reduction in both MOC2-399 and MOC2-400 tumors (Figure 2B), while CD8 T cell levels had a statistically significant increase in MOC2-399 tumors (Figure 2G).

**Figure 3:**
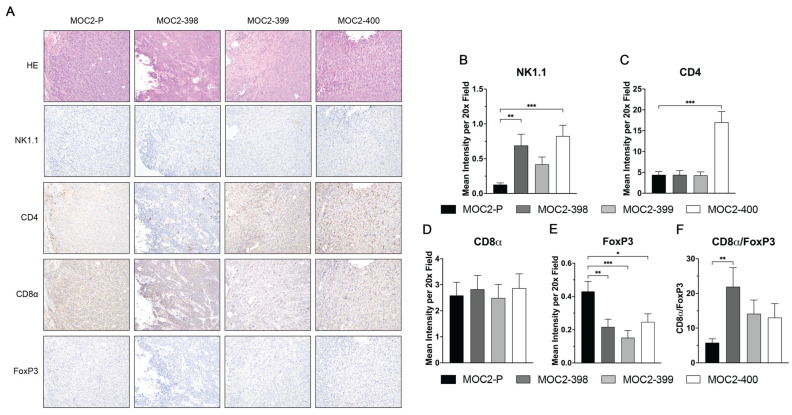
Axl KO results in increased NK cell infiltration. Subcutaneous tumors were generated in syngeneic (C57BL/6) mice using the MOC2 parental and Axl KO cell lines. (**A**–**F**) Tumors collected 27 days after inoculation and were stained using H&E and IHC. (**A**) Representative images for each group are shown (20× magnification). (**B**–**F**) Image quantification was performed, and the CD8α/FoxP3 ratio was calculated. Mean values and SEMs are shown (*n* = 18–24 images per group). * *p* < 0.05; ** *p* < 0.01; *** *p* < 0.001.

IHC was used to further assess the impact of Axl KO on the TIME (Figure 3A). Consistent with the flow cytometry results, IHC revealed increased NK cell infiltration in MOC2-398 and MOC2-400 tumors (Figure 3B). The levels of CD8a+ T cells remained unchanged upon Axl KO (Figure 3D), while T_reg_s (FoxP3+) levels were significantly decreased in all 3 Axl KO tumors (Figure 3E). Consequently, the CD8a/FoxP3 ratio was elevated in Axl KO tumors, although this was only statistically significant in MOC2-398, indicating a shift towards a more anti-tumor immune profile (Figure 3F). Additionally, the Axl knockout in each clone was confirmed to be stably maintained in vivo by IHC (Supplemental Figure S2).

Taken together, these results indicate that the slower growth of Axl KO tumors in vivo is not due to changes in tumor cell proliferation but is instead a result of significant alterations in the composition of the TIME. Axl KO enhances recruitment of NK cells while reducing the presence of T_reg_s, PMN-MDSCs, and other immunosuppressive cells, thereby creating a more pro-inflammatory TIME overall.

### 3.3. Tumor Growth Delay in Axl KO Cell Lines Is Mediated by Immune Cell Populations

**Figure 4:**
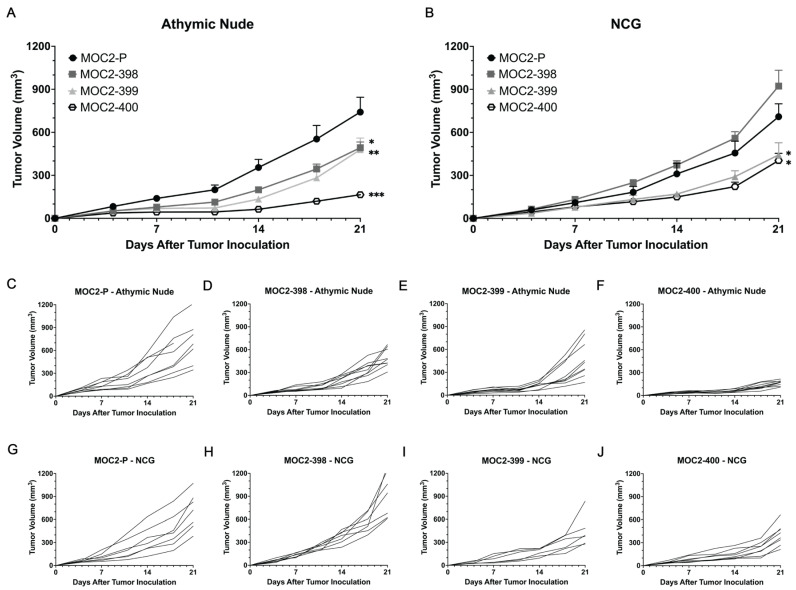
Tumor growth delay in Axl KO is mediated by immune cell populations. (**A**) Subcutaneous tumors were generated by inoculating 0.5 × 10^6^ cells/tumor in athymic nude mice. Tumor volume was measured twice per week. Mean values and SEMs are shown (*n* = 8 tumors per group). * *p* < 0.05; ** *p* < 0.01; *** *p* < 0.001. (**C**–**F**) Individual tumor growth rates are shown. (**B**) Subcutaneous tumors were generated by inoculating 0.5 × 10^6^ cells/ tumor in NCG mice. Tumor volume was measured twice per week. Mean values and SEMs are shown (*n* = 8 tumors per group). * *p* < 0.05. (**G**–**J**) Individual tumor growth rates are shown.

To further determine whether the tumor growth delay observed in the Axl KO cell lines was immune cell mediated, we examined specific immune cell populations potentially driving this phenotype. Given the broad range of immune cell populations altered by Axl KO, we aimed to identify key contributors by utilizing immune-deficient mouse models. While the syngeneic mouse models used in Figure 1 retain all immune cell populations, athymic nude mice lack T cells, and NCG mice lack T cells, B cells, and NK cells (Figure 4). Interestingly, the tumor growth delays observed in Axl KO tumors were similar in both syngeneic mice (Figure 1B) and athymic nude mice (Figure 4A,C–F), suggesting that T cells may not play a significant role in the growth delay caused by Axl KO. However, in NCG mice, Axl KO tumors exhibited different growth patterns (Figure 4B,G–J). Specifically, MOC2-398 tumors grew at rates comparable to MOC2-P tumors, while MOC2-400 tumors displayed a less pronounced growth delay than in syngeneic models. These results suggest that the tumor growth delay observed in Axl KO tumors may be influenced by NK cell and/or B cell activity within the TIME. As expected, MOC2-P tumor growth was not significantly impacted by the absence of T cells, NK cells, or B cells, consistent with the highly immune evasive nature of MOC2 tumors [30]. Together, these data indicate that the tumor growth delay observed in Axl KO cell lines is likely mediated by NK cells and/or B cells, highlighting the role of Axl in regulating immune cell activity within the TIME.

### 3.4. Axl KO Changes Expression of Immunomodulatory Cytokines and Chemokines

**Figure 5:**
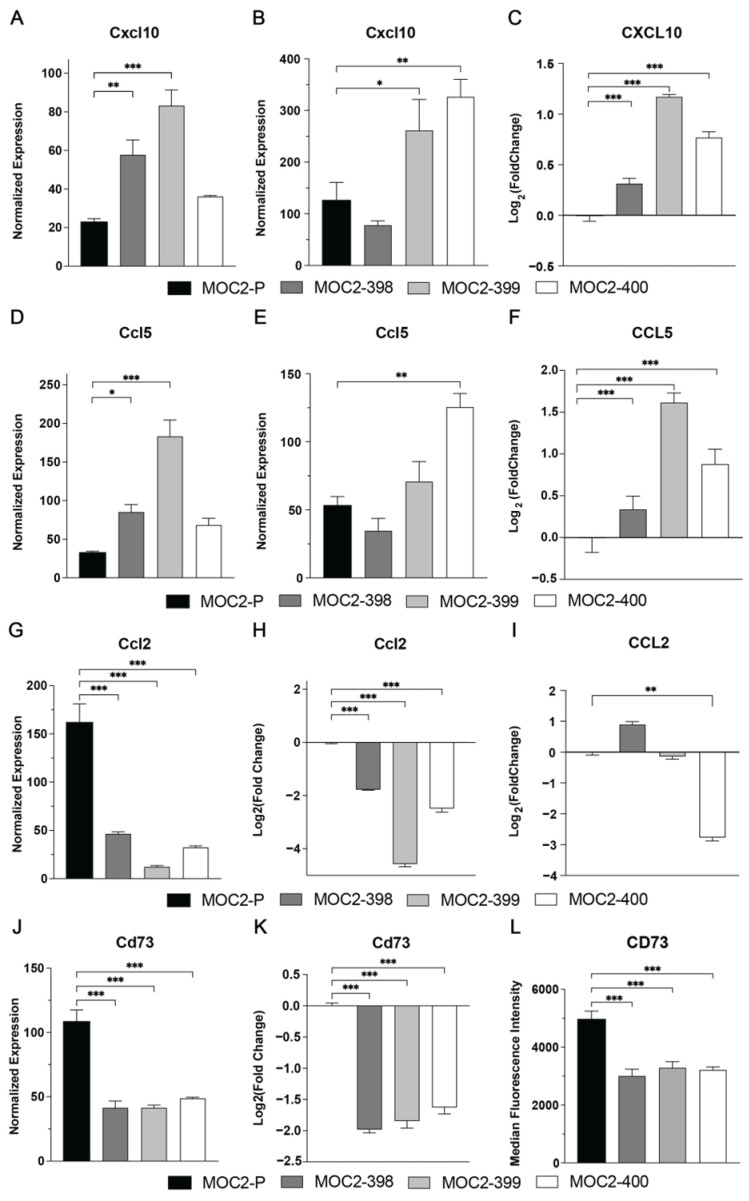
Axl KO impacts modulators of NK cell recruitment and cytotoxicity. (**A**,**D**,**G**,**J**) RNA was collected from cultured cells, and the expression level of the indicated genes was determined by the NanoString nCounter Tumor Signaling 360 panel. Means and SEMs are shown (*n* = 3 per group). * *p* < 0.05; ** *p* < 0.01; *** *p* < 0.001. (**B**,**E**) RNA was collected from subcutaneous tumors grown in syngeneic (C57BL/6) mice, and the expression level of the indicated genes was determined by the NanoString nCounter PanCancer Immune Profiling panel. Means and SEMs are shown (*n* = 3 per group). * *p* < 0.05; ** *p* < 0.01. (**C**,**F**,**I**) Supernatant was collected from cell culture plates after 24 h, and the level of the indicated proteins was determined by a Luminex ProCartaPlex panel. Means and SEMs are shown (*n* = 9 per group). ** *p* < 0.01; *** *p* < 0.001. (**H**,**K**) RNA was collected from cultured cells, and the expression level of the indicated genes was determined by qPCR. Mean values and SEMs are shown (*n* = 3 per group). *** *p* < 0.001. (**L**) Cultured cells were analyzed via flow cytometry. *** *p* < 0.001.

We next investigated the mechanism by which Axl modulates immune cell recruitment and activity utilizing NanoString, qPCR, Luminex, and flow cytometry. These analyses identified four key immunomodulatory factors affected by Axl KO: CXCL10, CCL5, CCL2, and CD73 (Figure 5). CXCL10, a chemokine critical for NK cell recruitment and activity [31,32], was significantly increased in Axl KO cell lines, as demonstrated by in vitro NanoString nCounter for MOC2-398 and MOC2-399 (Figure 5A)*,* in vivo NanoString nCounter for MOC2-399 and MOC2-400 (Figure 5B), as well as in vitro Luminex analysis for all three Axl KOs (Figure 5C). Similarly, the expression and secretion of CCL5, a chemokine known to promote NK cell recruitment [33] and enhance NK cell cytotoxicity [34], was also significantly upregulated in Axl KO cell lines as demonstrated by in vitro NanoString nCounter for MOC2-398 and MOC2-400 (Figure 5D), in vivo NanoString nCounter for MOC2-399 (Figure 5E), along with in vitro Luminex analysis for all 3 Axl KOs (Figure 5F).

In contrast, Axl KO led to a reduction in the expression and secretion of CCL2 and CD73. CCL2 is a chemokine associated with MDSC and macrophage recruitment, both of which are known to strongly inhibit NK cell activity through a variety of mechanisms [13]. The observed decrease in CCL2 following Axl KO, as demonstrated by in vitro NanoString nCounter for all 3 Axl KOs (Figure 5G), in vitro qPCR for all 3 Axl KOs (Figure 5H), and in vitro Luminex analysis for MOC2-400, likely enhances NK cell functionality. Additionally, CD73, an ectonucleotidase that converts adenosine monophosphate (AMP) into adenosine, was significantly downregulated in all 3 Axl KO cell lines as demonstrated by in vitro NanoString nCounter (Figure 5J), in vitro qPCR (Figure 5K), and in vitro flow cytometry (Figure 5L). Given that adenosine strongly suppresses NK cell cytotoxicity [13,35,36], the reduction in CD73 further supports a mechanism by which NK cell activity is enhanced in the absence of Axl. We also observed a decrease in PDL1 and CD276 expression (Supplemental Figure S3) in Axl KO cell lines, both of which are associated with impaired NK cell activity and cytotoxicity [37,38]; however, these changes were only observed upon in vitro flow cytometry analysis. Collectively, these findings suggest that Axl KO alters the chemokine and receptor landscape within MOC2 tumors by increasing factors that promote NK cell recruitment and cytotoxicity and by decreasing factors that suppress NK cell activity.

### 3.5. Axl KO Enhances NK Cell Cytotoxicity and Recruitment

**Figure 6:**
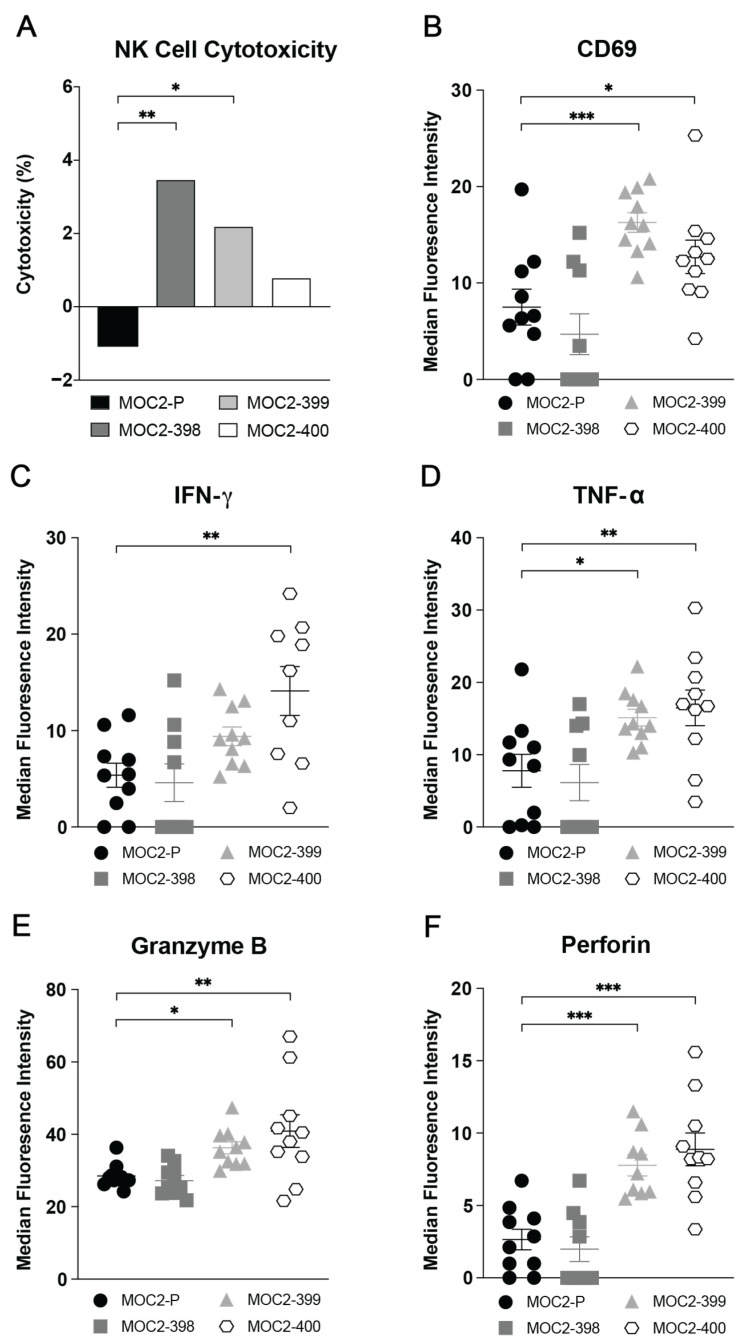
Axl KO enhances NK cell cytotoxicity. (**A**) Primary NK cells (1 × 10^5^ per well) were cultured with MOC2 parental and Axl KO cell lines (1 × 10^4^ per well) for 4 h. Lactate dehydrogenase (LDH) was measured, and percent cytotoxicity was calculated. * *p* < 0.05; ** *p* < 0.01. Subcutaneous tumors were generated in syngeneic (C57BL/6) mice by inoculating 0.5 × 10^6^ MOC2 parental and Axl KO cells per tumor. Tumors were collected 24 days after inoculation, and expression levels of (**B**) CD69, (**C**) IFN-γ, (**D**) TNF-α, (**E**) Granzyme B, and (**F**) Perforin on or within tumor-infiltrating NK cells were analyzed via flow cytometry. Mean and SEMs are shown (*n* = 9–10 per group). * *p* < 0.05; ** *p* < 0.01; *** *p* < 0.001.

Building on our findings that Axl KO alters the expression of key chemokines and receptors that enhance NK cell recruitment and activity (Figure 5), we sought to determine the functional significance of these changes. While both B cells and NK cells were implicated in mediating the tumor growth delay observed in Axl KO tumors (Figure 4), prior work from our laboratory has shown that pharmacologic inhibition of Axl increases NK cell activation [21]. Therefore, we tested the hypothesis that Axl KO enhances NK cell activity and cytotoxicity, contributing to the observed tumor growth delay. We first assessed NK cell cytotoxicity in vitro using primary NK cell co-culture assays with parental and Axl KO MOC2 cell lines. As expected, NK cells exhibited significantly enhanced cytotoxic activity against MOC2-398 and MOC2-399 Axl KO tumor cells compared to MOC2-P (Figure 6A).

To determine if this increase in NK cell cytotoxicity occurs in vivo, we evaluated markers of activation and cytotoxicity in parental and Axl KO tumor infiltrating NK cells via flow cytometry analysis. Notably, NK cells infiltrating MOC2-399 and MOC2-400 Axl KO tumors exhibited higher expression of the activation marker CD69 (Figure 6B) [39,40]. Additionally, these infiltrating NK cells in Axl KO tumors showed significantly elevated expression of the pro-inflammatory cytokines IFN-γ in MOC2-400 (Figure 6C) and TNF-α in MOC2-399 and MOC2-400 (Figure 6D), which play critical roles in signaling other cytotoxic immune cells and inducing apoptosis in target cells [41,42]. Furthermore, NK cells infiltrating Axl KO tumors demonstrated enhanced cytotoxic potential, as shown by increased expression of granzyme B in (Figure 6E) and perforin in MOC2-399 and MOC2-400 (Figure 6F), both of which are responsible for NK cell-mediated killing [43].

**Figure 7:**
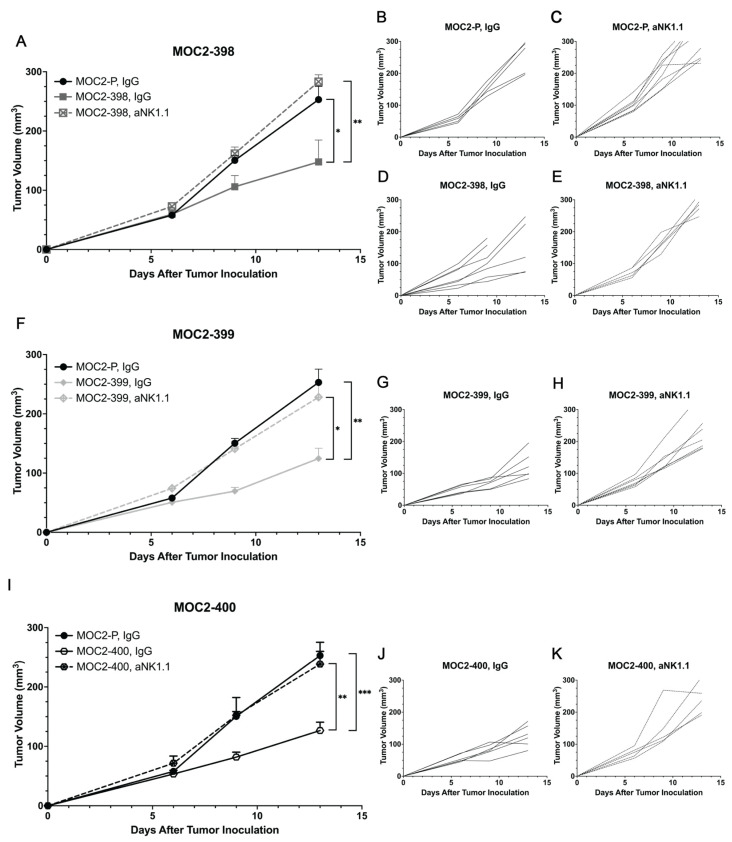
NK cell depletion rescues Axl tumor growth. (**A**,**F**,**I**) Subcutaneous tumors were generated in syngeneic (C57BL/6 mice). Tumor volume was measured twice per week. Mean values and SEMs are shown (*n* = 8 per group). * *p* < 0.05; ** *p* < 0.01; *** *p* < 0.001. Tumor-bearing mice were treated with 50 μg IgG or 50 μg anti-NK1.1 every 5 days (Day −5, 0, 5, 10). Tumor volume data for MOC2-P, IgG group is presented on each graph. (**B**–**E**,**G**,**H**,**J**,**K**) Individual tumor growth rates are shown.

To further assess the impact of NK cells in vivo, we implanted MOC2-P (Figure 7A–C,F,I) MOC2-398 (Figure 7A,D,E), MOC2-399 (Figure 7F–H), and MOC2-400 (Figure 7I–K) into syngeneic mice and depleted NK cells using an anti-NK1.1 (aNK1.1) antibody. Tumor growth of each Axl KO cell line was then individually compared to MOC2-P tumor growth. Strikingly, NK cell depletion completely rescued tumor growth in each of the three Axl KO clones, with aNK1.1-treated Axl KO tumors growing similarly to the parental (MOC2-P) tumors. NK cell depletion was confirmed in the spleens and tumors of the mice (Supplemental Figure S4). These results strongly suggest that enhanced NK cell activity, recruitment, and cytotoxicity are key drivers of the tumor growth delay seen in Axl KO tumors. Collectively, these data highlight the pivotal role of Axl in mediating the activity of NK cells within the HNC TIME.

## 4. Discussion

The receptor tyrosine kinase Axl has long been linked to solid tumor progression, metastasis, and therapeutic resistance. In recent years, the role of Axl as a regulator of the tumor immune microenvironment has begun to be uncovered, but much about the specific mechanism remains unknown. This report presents data suggesting that Axl signaling promotes tumor growth and a colder TIME in HNC by excluding NK cells via alterations in the expression profile of specific chemokines and receptors.

The impact of Axl signaling on solid tumor growth is well established; studies by our laboratory and others have shown that Axl targeting, either genetically or pharmacologically, significantly slows tumor progression in many cancer types, including head and neck [15,21], breast [26,44,45,46,47], ovarian [23,48], lung [49,50], pancreatic [24], bladder [26], colon [47], and skin cancer [51]. The current study supports these findings, showing that Axl KO slows tumor growth in a mouse model of HNC (Figure 1B). Further, we demonstrated that this change in tumor growth was not due to changes in tumor proliferation rate (Figure 1G–J), suggesting that Axl is influencing the composition of the immune cells in the TIME.

Axl’s role in the regulation of the immune system is also well known. A previous study by our laboratory showed that simultaneous inhibition of Axl and its family member MerTK creates a hotter TIME in HNC [21]. Other studies also support Axl’s role in modulating the immune response, though the exact mechanism remains unclear [20,22,23,24,26,44,46,51,52,53,54,55]. However, most studies agree that disruption of Axl signaling results in a more pro-inflammatory TIME, a finding that is supported by our current study. We showed in Figure 2 and Figure 3 that Axl KO tumors have lower levels of infiltrating immunosuppressive cells and higher levels of infiltrating pro-inflammatory cells, demonstrating that Axl KO creates a hotter TIME.

Specifically, we observed increased levels of NK cells and decreased levels of PMN-MDSCs (Figure 2B), B cells (Figure 2F), and T_reg_s (Figure 2E,J–L) in Axl KO tumors. Other studies have noted the impact of Axl on some of these cell types. A study in a mouse model of breast cancer found that Axl KO tumors were significantly enriched for NK cells and had fewer T_reg_s than wild-type tumors [56]. In another breast cancer model, Axl KO was associated with increased CD8 T cell infiltration, though T_reg_ levels did not appear to change significantly [20]. MDSCs have also been shown to be impacted by the TAMs; one study in a mouse model of melanoma demonstrated that TAM KO significantly reduced the suppressive capacity of MDSCs [51]. Pharmacologic inhibition of Axl has been shown to have similar effects on immune cells. Treatment with a pan-TAM inhibitor, RXDX-106, significantly increased the level of NK cell infiltration in a mouse model of colon cancer [47], and treatment with the Axl inhibitor R428 was able to sensitize lung cancer cells to NK cell-mediated cytotoxicity [54]. The report by Terry et al., showing that Axl inhibition can increase cancer cells’ susceptibility to NK cell-mediated lysis [54], is consistent with the current study’s findings. Our data suggest that Axl regulates NK cell recruitment and cytotoxicity to create a cold TIME and promote tumor growth (Figure 6 and Figure 7).

Finally, we present data that suggest potential mechanisms for the Axl-mediated effects on NK cells (Figure 5). Expression of the chemokine CXCL10 was significantly increased upon Axl KO. CXCL10 is a well-known chemoattractant for NK cells [31,32], and Axl inhibition has previously been shown to result in increased CXCL10 expression [23]. In HNC, CXCL10 expression has been positively correlated with overall survival [57], and studies in other cancers have identified CXCL10 as a positive prognostic factor for response to immunotherapy [58,59]. CCL5, another chemokine associated with NK cell recruitment [33] and cytotoxicity [34], was also increased with Axl KO. However, Axl inhibition with small molecule inhibitors has been shown to result in lowered CCL5 expression [20,23,24]. This contradiction may be explained by Axl’s expression on many immune cell types [60]. In the model presented here, Axl is only absent from the cancer cells, but systemic pharmacologic inhibition impacts Axl wherever it is expressed, in cancer cells and immune cells alike. Further research investigating Axl-mediated regulation of CCL5 expression is needed.

Expression of another chemokine, CCL2, was significantly decreased in the Axl KO cell lines (Figure 5). CCL2 acts as a chemoattractant for MDSCs and macrophages [61,62,63,64], both of which have been shown to inhibit NK cells in HNC [13]. Previous studies have demonstrated that genetic and pharmacologic targeting of Axl decreases expression and secretion of CCL2 in mouse cancer models [23,56]. Finally, expression of the ectonucleotidase CD73 was significantly decreased upon Axl KO. CD73 converts AMP into the immunosuppressive molecule adenosine, which inhibits NK cells and other pro-inflammatory immune cells [13,35,36]. Specifically, adenosinergic signaling negatively affects NK cell cytokine production, cytotoxicity, and activating receptor expression [65]. High levels of Axl expression have been positively associated with CD73 expression [66,67], and interestingly, CD73 knockdown has been shown to result in a decrease in Axl expression [68].

While the data presented here suggest Axl is responsible for disruption of NK cell recruitment and function leading to tumor growth and immune evasion, other immune cell types may be involved. CXCL10 and CCL5 serve to attract more than just NK cells; CXCL10 is a chemoattractant for T cells, eosinophils, and monocytes [69], and CCL5 recruits T cells, DCs, mast cells, eosinophils, basophils, and monocytes [70]. MDSCs could also be involved, as the data show a decrease in infiltrating MDSCs and downregulation of the MDSC chemoattractant CCL2 upon Axl KO (Figure 2 and Figure 5). Further research into the impact of genetic or pharmacologic Axl targeting on these cell types in HNC is needed.

A limitation of this study is the clonal heterogeneity that the Axl KO cell lines display. This heterogeneity could have resulted from differences in adaptation or compensatory pathways as a result of Axl KO, which is commonly seen in CRISPR-Cas9 edited cell lines [71]. Although trends varied slightly between the Axl KO cell lines, our key findings were observed in at least two of the Axl KO clones, including increased NK cell infiltration, upregulation of NK cell recruiting chemokines, and enhanced NK cell cytotoxicity. Additionally, some of the statistically significant changes in immune cell populations observed were only slight decreases or increases in percentages, and further testing would be needed to confirm the biological relevance of these changes. We do conclude, however, that the 1–3% increase in NK cells in Axl KO tumors contributes to a functionally relevant anti-tumor immune response and delayed tumor growth given the increase in NK cell cytotoxicity in Axl KO tumors and Axl KO tumor growth being “rescued” upon NK cell depletion. While the trends observed remained consistent across three Axl KO clones, this study is somewhat limited due to the use of only one mouse HNC cell line (MOC2). More work is needed to further confirm the relationship between Axl and NK cell modulation in other HNC mouse models as well as in human HNC patients.

The current study demonstrates that Axl signaling significantly alters the growth of HNC tumors by promoting an immunosuppressive TIME via the disruption of NK cell recruitment and cytolytic function. As our group has previously demonstrated with the dual inhibition of Axl and MerTK in combination with PD-L1 inhibition in pre-clinical mouse models, targeting Axl could potentially improve the efficacy of other ICIs, such as anti-CTLA4. However, further studies are needed to confirm this in both pre-clinical and clinical models [21]. NK cell therapies are increasingly being developed for cancer treatment [72,73,74], and as these therapies begin to be utilized for patients with solid tumors, understanding the mechanisms of cancer cell-mediated NK inhibition is critical. Because Axl is so widely expressed in HNC (over 80% of HNC tumors are positive for Axl expression [15,75]), combining NK cell therapies, such as CAR-NK cells [76,77] and NK cell engagers [78], with simultaneous Axl inhibition may be required for optimal clinical efficacy and could potentially lead to more positive patient outcomes.

## 5. Conclusions

Results from this study highlight the critical role of the receptor tyrosine kinase Axl in contributing to an immunosuppressive TIME in HNC. Genetic knockout of tumor-bound Axl in an HNC mouse model significantly delayed tumor growth, primarily through alterations in immune cell populations infiltrating the tumors. Specifically, Axl KO tumors exhibited a marked increase in activated NK cell infiltration in vivo, along with elevated levels of key immunomodulators, such as CXCL10 and CCL5, which are known to recruit and activate NK cells. Additionally, NK cells infiltrating Axl KO tumors displayed enhanced cytotoxicity as shown by increased production of cytotoxic proteins, including IFN-gamma and granzyme B. Importantly, the delayed tumor growth observed in Axl KO tumors was reversed upon NK cell depletion, underscoring the pivotal role of NK cells in mediating this effect. These results suggest that Axl modulates NK cell recruitment and activity within the TIME and that therapeutically targeting Axl, particularly in combination with immunotherapies, holds significant potential to enhance treatment responses in HNC patients.

## Data Availability

The datasets used and/or analyzed during the current study are available from the corresponding author on reasonable request.

## Supplementary Materials

The following supporting information can be downloaded at: https://www.mdpi.com/article/10.3390/cancers17060994/s1, Table S1: Immunoblot antibodies; Table S2: Flow cytometry antibodies; Table S3: Flow cytometry gating paths; Table S4: TaqMan probes; Table S5: IHC antibodies; Figure S1: Validation of Axl KO cell lines; Figure S2: Axl KO cell line maintains Axl loss in vivo; Figure S3: Axl KO impacts immunomodulators; Figure S4: NK cell depletion confirmation; Figure S5: Uncropped Western Blot images for Figure 1A.

## Abbreviations

The following abbreviations are used in this manuscript:

Axl	Receptor tyrosine kinase UFO
HNC	Head and neck cancer
HPV	Human papillomavirus
TIME	Tumor immune microenvironment
RTK	Receptor tyrosine kinase
PDX	Patient-derived xenograft
NK	Natural killer cell
KO	Knockout
MOC	Mouse oral cancer
FBS	Fetal bovine serum
RNP	Ribonucleoprotein
gRNA	Guide RNA
PCR	Polymerase chain reaction
PMN-MDSCs	Polymorphonuclear myeloid-derived suppressor cells
IHC	Immunohistochemistry
