# Supplementary material for: Axl Regulation of NK Cell Activity Creates an Immunosuppressive Tumor Immune Microenvironment in Head and Neck Cancer
Source: Cancers (Basel). 2025 Mar 15;17(6):994. doi: 10.3390/cancers17060994 (PMC11940164; doi:10.3390/cancers17060994)
Supplement: Supplementary file 1 [file cancers-17-00994-s001.zip › Supplemental Tables and Figures.pdf]

Supplemental Table S1: Immunoblot Antibodies

Antigen	Vendor	Catalog Number
Axl	Proteintech	13196-1-AP
α-Tubulin	Millipore Sigma	CP-06

Supplemental Table S2: Flow Cytometry Antibodies

Antigen	Fluorescent Marker	Vendor	Catalog Number
Axl	APC	Thermo Fisher Scientific	17-1084-82
CD117	Alexa Fluor 700	Biolegend	105846
CD11b	PE-Vio615	Miltenyi Biotec	130-113-807
CD11c	Super Bright 600	Thermo Fisher Scientific	63-0114-82
CD170	BB515	BD Biosciences	566211
CD19	Super Bright 702	Thermo Fisher Scientific	67-0193-80
CD206	APC	Biolegend	141708
CD25	Brilliant Violet 510	Biolegend	102041
CD27	PE-Vio615	Miltenyi Biotec	130-123-043
CD276	Real Yellow 586	BD Biosciences	753240
CD3	Alexa Fluor 488	Biolegend	100210
CD3	Brilliant Violet 510	Biolegend	100234
CD4	PerCP-eFluor 710	Thermo Fisher Scientific	46-0041-82
CD45	Brilliant Violet 711	Biolegend	103147
CD45	PE-Cy7	Biolegend	103114
CD45	BUV 395	Thermo Fisher Scientific	363-0451-80
CD69	PE-Cy5	Biolegend	104509
CD73	Nova Fluor Yellow 690	Thermo Fisher Scientific	M032T02Y05
CD8α	Alexa Fluor 700	Biolegend	100730
CD80	Super Bright 436	Thermo Fisher Scientific	62-0801-82
Cytokeratin 18	Alexa Fluor 488	Thermo Fisher Scientific	MA5-18157
F4/80	Super Bright 702	Thermo Fisher Scientific	67-4801-82
FcεR1α	PerCP-eFluor 710	Thermo Fisher Scientific	46-5898-82
FoxP3	PE	Tonbo Biosciences	50-5773-U100
Granzyme B	PE-Dazzle 594	Biolegend	372216
IFN-γ	PE-Cy7	Thermo Fisher Scientific	25-7311-82
Live/Dead	Ghost Red 780	Tonbo Biosciences	13-0865-T100
Ly6C	PE-Cy5	Elabscience	E-AB-F1121UG
Ly6G	PE	Tonbo Biosciences	50-1276-U025
MHCII (I-A/I-E)	Brilliant Violet 510	Biolegend	107636
MHCII (I-Ab)	Brilliant Violet 510	BD Biosciences	744929
NK1.1	Super Bright 436	Thermo Fisher Scientific	62-5941-82
PD1	Super Bright 600	Thermo Fisher Scientific	63-9985-82
PDL1	PE-Cy7	Biolegend	155406
PDL1	PE-Cy7	Tonbo Biosciences	60-1243-U100
Perforin	APC	Biolegend	154303
TNF-α	Brilliant Violet 786	Thermo Fisher Scientific	417-7321-82

Supplemental Table S3: Flow Cytometry Gating Paths

Cell Type	Gating Path[a]
CD4 T Cells	Singlets/Live/CD45+/CD3+/CD4+, CD8-
CD8 T Cells	Singlets/Live/CD45+/CD3+/CD4-, CD8+
Classic Tregs	Singlets/Live/CD45+/CD3+/CD4+, CD8-/FoxP3+, CD25+
Immature Tregs	Singlets/Live/CD45+/CD3+/CD4+, CD8-/FoxP3+, CD25-
NK Cells	Singlets/Live/CD45+/CD3-/NK1.1+
B Cells	Singlets/Live/CD45+/CD3-/CD19+
Exhausted CD8 T Cells	Singlets/Live/CD45+/CD3+/CD4-, CD8+/PD1+
Neutrophils	Singlets/Live/CD45+/CD11b+/F480+/CD11c-, MHCII-/CD170-/Ly6G+
PMN-MDSCs	Singlets/Live/CD45+/CD11b+/F480-/CD11c-, MHCII-/Ly6G+/Ly6C-
Plasmacytoid DCs	Singlets/Live/CD45+/CD11b-/CD11c+/MHCII low/Ly6C+
TILs	Singlets/Live/CD45+
Total Tregs	Singlets/Live/CD45+/CD3+/CD4+, CD8-/FoxP3+ OR CD25+
Tumor Cells	Singlets/Live/CD45-/Cytokeratin18+

[a]Gates separated by a slash (/) indicate a parent/child relationship. Gates separated by a comma (,) indicate a quadrant gate relationship.

Supplemental Table S4: TaqMan Probes

Gene	Assay ID
Ccl2	Mm00441242_m1
Cd73	Mm00501910_m1
Gapdh	Mm99999915_g1
β-actin	Mm00607939_s1

Supplemental Table S5: IHC Antibodies

Antigen	Vendor	Catalog Number
Axl	Proteintech	13196-1-AP
CD8α	Thermo Fisher Scientific	14-0808-82
CD4	Thermo Fisher Scientific	14-9766-82
FoxP3	Thermo Fisher Scientific	14-5773-82
NK1.1	Cell Signaling Technology	39197
Ki67	Cell Signaling Technology	12202

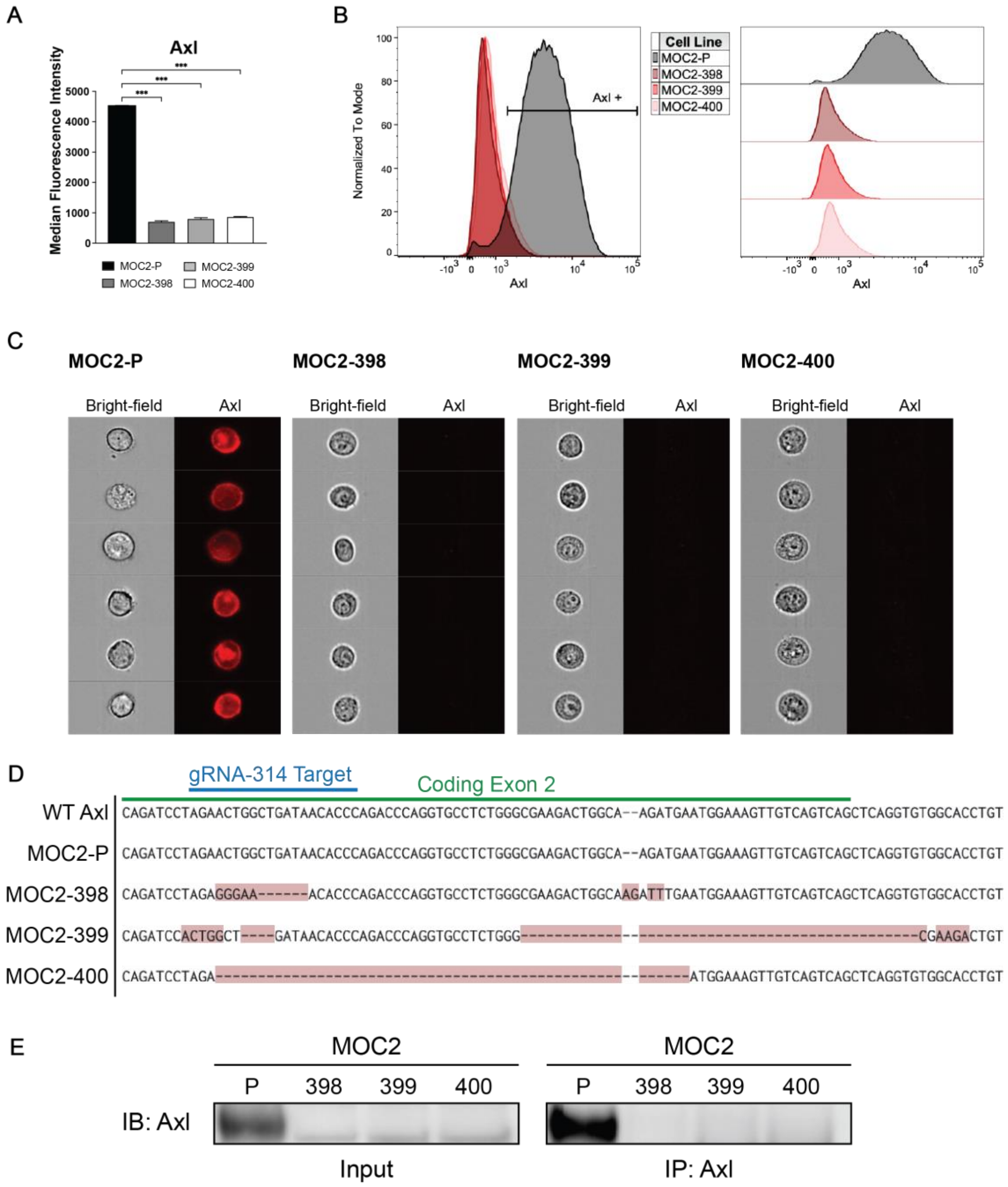

Figure S1: Validation of Axl KO cell lines

(A-B) Cultured cells were analyzed via flow cytometry. Mean values and SEMs are shown ($n = 3$ per group) and are representative of three independent experiments. ***, $P < 0.001$. (C) Cultured cells were analyzed via imaging flow cytometry. Representative images for each group are shown (40X magnification) (D) DNA was collected from cultured cells and sequenced, then compared to the wild-type (WT) Axl sequence. Red highlights indicate insertions/deletions. (E) Whole cell lysates were harvested and 500µg of lysate was subjected to immunoprecipitation with anti-Axl. The original whole cell lysate (Input) and the immunoprecipitated lysate (IP:Axl) was fractionated via SDS-PAGE, followed by immunoblotting for Axl.

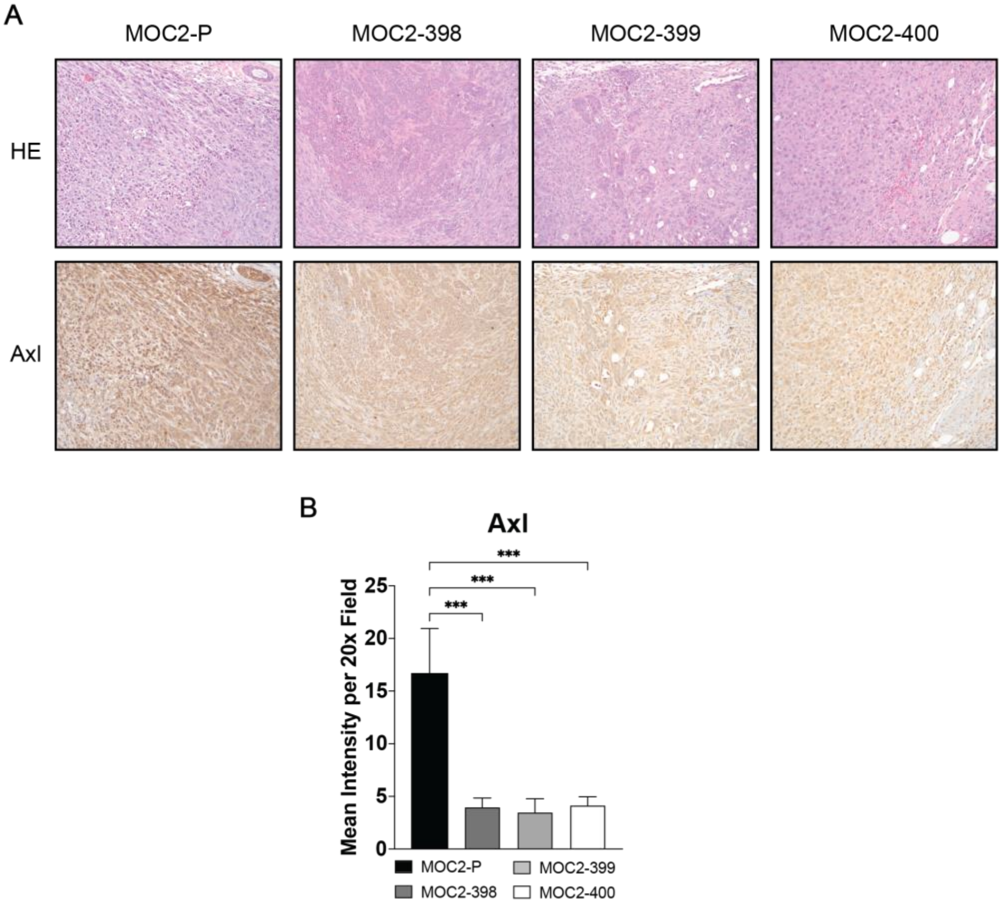

Figure S2: Axl KO cell line maintains Axl loss *in vivo*

Subcutaneous tumors were generated in syngeneic (C57BL/6) mice. Tumors were collected after 27 days and stained using H&E or IHC (A, B) Representative IHC images for each group (20X magnification) are shown. Image quantification was performed (n=15 images per group). *** $P < 0.001$.

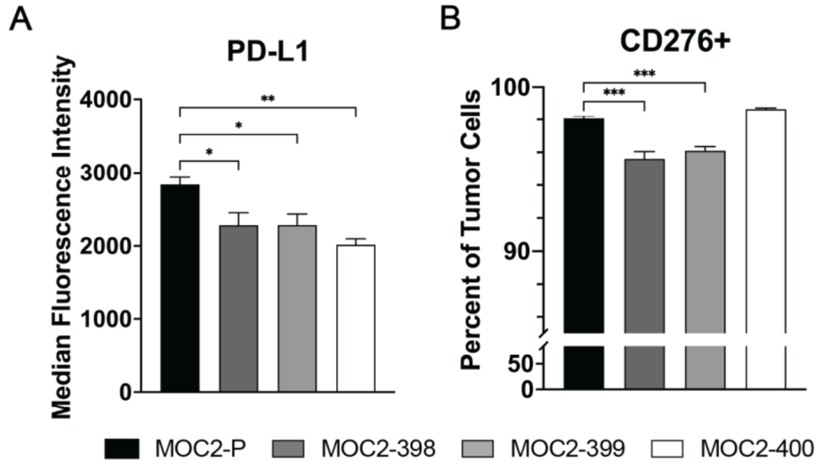

Figure S3: Axl KO impacts immunomodulators
(A, B) Cultured cells were analyzed via flow cytometry. Mean values and SEMs are shown ($n = 3$ per group). * $P < 0.05$; ** $P < 0.01$; *** $P < 0.001$.

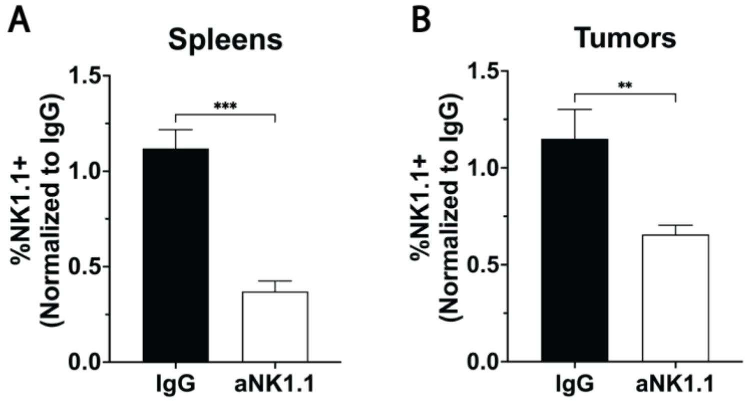

Figure S4: NK cell depletion confirmation
Using an antibody against NK1.1, NK cells were depleted from C57BL/6 mice. Mice were treated with 50μg anti-NK1.1 or IgG every five days (Day -5, 0, 5, 10, for 18 days, totaling 4 treatments. (A) Spleens (n=4 per group) and (B) tumors (n=4 per group) were collected, and tissue-infiltrating NK cells were analyzed by flow cytometry. Mean percentage of NK cells, normalized to the IgG group, and SEMs are shown ($n = 32$ per group). ** $P < 0.01$; *** $P < 0.001$.
