# Supplementary figures and images for: Axl Regulation of NK Cell Activity Creates an Immunosuppressive Tumor Immune Microenvironment in Head and Neck Cancer
Source: Cancers (Basel). 2025 Mar 15;17(6):994. doi: 10.3390/cancers17060994 (PMC11940164; doi:10.3390/cancers17060994)

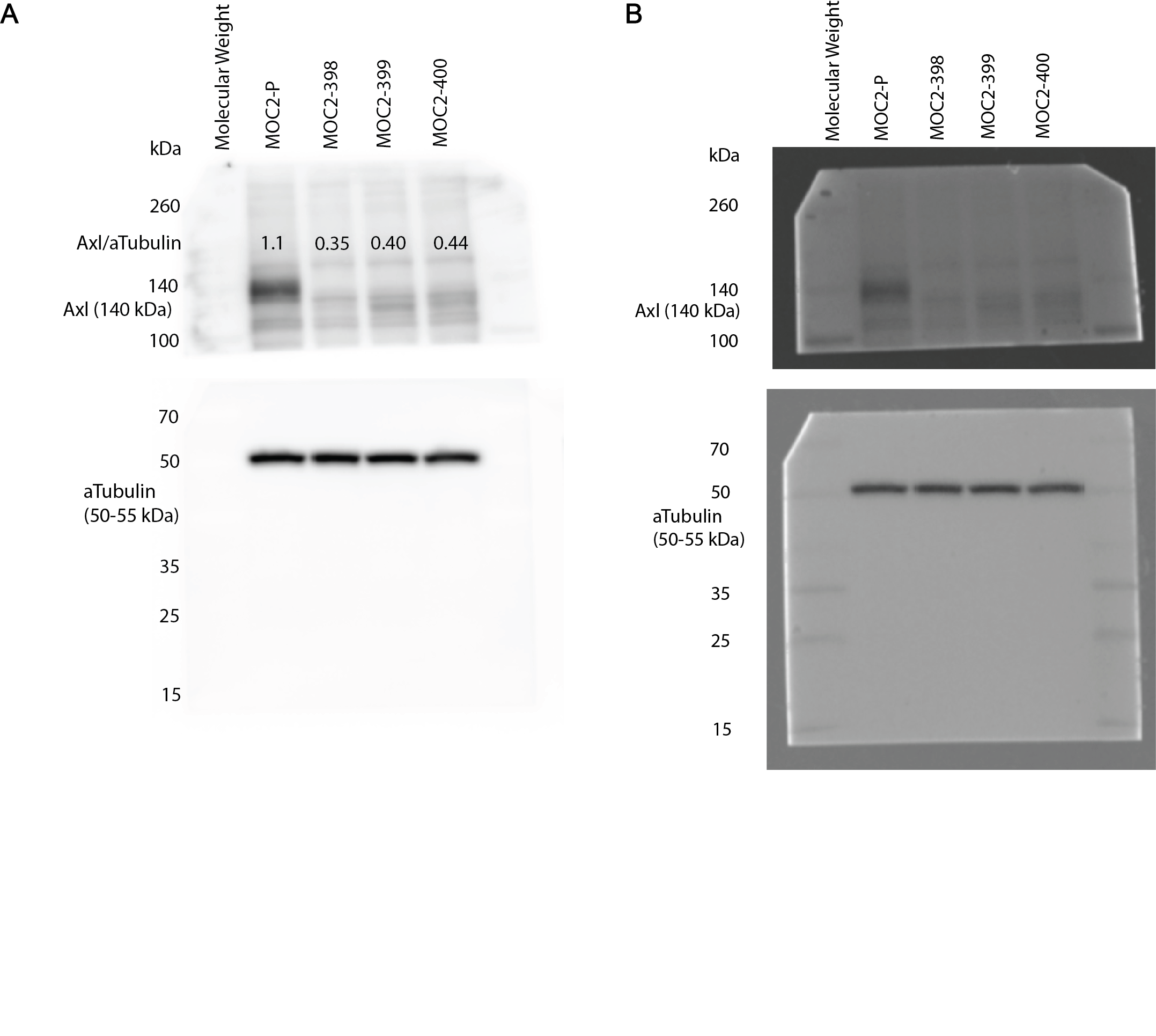

Supplement: Supplementary file 1 [file cancers-17-00994-s001.zip › Figure S5. Uncropped Western Blot images.png]
